# Saponin and Phenolic Composition and Assessment of Biological Activities of *Saponaria officinalis* L. Root Extracts

**DOI:** 10.3390/plants13141982

**Published:** 2024-07-19

**Authors:** Despina Charalambous, Michalis Christoforou, Krystallo Christou, Melina Christou, Antonis Ververis, Marios Andreou, Kyproula Christodoulou, Andrie Koutsoulidou, Christoforos Papachrysostomou, Maria Pantelidou

**Affiliations:** 1 Frederick Research Center, Nicosia 1036, Cyprus; mic.christoforou@gmail.com (M.C.); krystallo.christou@gmail.com (K.C.);; 2 Department of Pharmacy, School of Health Sciences, Frederick University, Nicosia 1036, Cyprus; 3 Molecular Genetics, Function & Therapy Department, The Cyprus Institute of Neurology and Genetics, 6 Iroon Avenue, P.O. Box 23462, Nicosia 1683, Cyprus; christoum@cing.ac.cy (M.C.); andriek@cing.ac.cy (A.K.); 4 Neurogenetics Department, The Cyprus Institute of Neurology and Genetics, Nicosia 2371, Cyprus; antonisv@cing.ac.cy (A.V.); roula@cing.ac.cy (K.C.); 5 Nature Conservation Unit, Frederick University, Nicosia 1036, Cyprus; m.andreou@frederick.ac.cy; 6 Veterinary Drug Residues Lab, State General Laboratory, Ministry of Health, Nicosia 2081, Cyprus; cpapachrysostomou@sgl.moh.gov.cy

**Keywords:** *Saponaria officinalis*, saponins, phenolics, antimicrobial, anticancer

## Abstract

The purpose of this study was to identify the saponin and phenolic components in root extracts of *Saponaria officinalis*, a widespread species, found in Cyprus. A total of six major saponins, including gypsogenin and gypsogenic acid derivatives, as well as saponariosides C, D, and E, were identified using UHPLC/Q-TOF-MS analysis, with gypsogenin derivatives being the most common saponins detected through quantitative analysis. A total of six phenolic compounds were also identified, including rutin, quercetin galactoside, syringic acid, apigenin, protocatechuic, and vanillic acid. In addition to their saponin and phenolic contents, the root extracts were prepared through different extraction methods, and their biological activity was assessed. All samples demonstrated antioxidant capacity, as well as antibacterial activity, against four bacterial strains (*Escherichia coli*, *Staphylococcus aureus*, *Enterococcus faecalis*, and *Salmonella enteritidis*), with the acetone extract presenting higher susceptibility. The evaluation of anticancer activity in A375 (human malignant melanoma), HeLa (human cervical epithelioid carcinoma), and HaCaT (healthy human keratinocytes) cell lines revealed that the acetone extract of *S. officinalis* extract demonstrated a significant inhibitory effect on the proliferation of A375 cells in a concentration-dependent manner. None of the extracts demonstrated anti-neurotoxic potential against Aβ_25–35_ cytotoxic peptides. The results of this study support previous findings that reveal that the *Saponaria* species are an excellent natural source of biologically active compounds with antioxidant, antimicrobial, and anticancer properties.

## 1. Introduction

*Saponaria officinalis* L., also known as common soapwort, is an ornamental plant from the Caryophyllaceae family. It is a common species in America, Europe, Asia, Africa, and Australia [1,2,3]. *S. officinalis* is an erect perennial plant, 30–90 cm high, with many branches, a stout axial root, and a fleshy, thin rhizome [2]. It has a simple or branched stem, usually glabrous, and ovate or ovate lanceolate leaves. Its flowers are sometimes double, with a green or reddish calyx, often cleft, having pink to white, often drying to dull, purple color. They are glabrous or, in rare occasions, with scattered trichomes [4,5]. The plant blooms from spring to fall and grows in waste places, streamsides, fields, and roadsides at an altitude up to 2600 m. It is a widely naturalized, sometimes troublesome weed. It may persist for years in abandoned home sites [4,5].

As its genus name implies and because of its high concentration of saponins, its roots can be used as a gentle cleanser when boiled in water. The plant’s extracts are used in the food and cosmetic industry because of the plant’s foaming and emulsifying properties [1]. *S. officinalis* has been traditionally used for its medicinal properties. Its roots and leaves are used to treat respiratory conditions such as bronchitis, coughs, and colds. The saponins in soapwort help to loosen mucus, thus facilitating its expulsion from the respiratory tract. Soapwort has been used topically to treat various skin conditions, including eczema, psoriasis, and acne, due to its cleansing and anti-inflammatory properties. Traditional medicine has also employed soapwort roots to promote urine production and to treat rheumatic pain [6]. Saponins are glycosides characterized by a structure containing a steroidal or triterpenoid aglycone and one or more sugar chains [1,2]. The saponins identified in *S. officinalis* include the saponariosides A–G, gypsogenin-based, and quillaic acid-based saponins. The saponariosides A–G are a series of glycosides derived from triterpenoid aglycones; gypsogenin-based and quillaic acid-based saponins are saponins with gypsogenin and quillaic acid as the aglycone part, respectively. *S. officinalis* has many diverse pharmacological effects due to the presence of saponins. Saponins from *S. officinalis* have shown significant anti-inflammatory effects that are mediated through the inhibition of cytokines [7]. They also exhibit antimicrobial properties against a variety of pathogens, including bacteria and fungi [6]. Some studies have reported the cytotoxic effects of saponins from *S. officinalis* on cancer cell lines, thus indicating their potential as antitumor agents [6].

In addition to saponins, the plant also contains flavonoids, phenolic compounds, and fatty acids [8,9]. The phenolic compounds produced in plants are divided into categories based on the number of phenolic rings which they contain, as well as other molecules attached to these rings. These secondary metabolites are known for their antioxidant [10], antimicrobial [11], anticancer [12], and neuroprotective activity [13]. Due to their rich content of phenolics, *S. officinalis* extracts have been reported to demonstrate antioxidant activity [14]. Their biological and, particularly, antibacterial activity have also been described in the literature [10,15,16].

This work aimed to determine the chemical composition of *S. officinalis* root extracts in terms of saponin and phenolic compounds. In addition to the identification of saponin and phenolic components, the antioxidant potential of the root extracts was assessed, as well as their antibacterial activity against Gram-positive and Gram-negative bacteria. Additionally, their anti-neurotoxic potential against Aβ_25–35_ cytotoxic peptides was tested. Lastly, the cytotoxicity of the extracts against cancer cell lines was determined.

## 2. Results

### 2.1. Identification and Quantification of Saponins in S. officinalis

The total saponin content (TSC) of *S. officinalis* root extracts was determined using three different solvents (methanol, ethanol, and acetone). A standard curve of oleanolic acid was constructed, and the results were expressed as mg oleanolic acid equivalents per gram of dry crude extract (mg OAE/g crude extract), as previously described [8]. According to the results, acetone gave the highest TSC yield (124.635 mg OAE/g crude extract), thus being significantly higher than the ethanol and methanol yields (103.117 and 9.535 mg OAE/g crude extract, respectively, *p* < 0.01) (Table 1).

The saponin compounds identified in the acetone *S. officinalis* root extracts, using ultrahigh-performance liquid chromatography coupled with quadrupole time of flight mass spectrometry (UHPLC-QTOF-MS), are presented in Table 2, and the total ion chromatogram is documented in the Supplementary Material (Figure S1). The MS/MS fragmentation patterns and chromatograms of each compound are presented in Figure S2. According to the results, six major saponins belonging to triterpene saponins were identified. These are glycosylated derivatives of triterpene sapogenin, which is the aglycone moiety of each compound. The mass spectrometric analysis of the saponin compounds allowed for the total identification of the compounds by direct comparison with previously published data on *S. officinalis* saponin fragmentation. Saponarioside E (compound **2**), saponarioside C and saponarioside D (compound **3**), and other saponins derived from gypsogenin (compounds **1** and **4**–**6**) were also identified (Table 1). According to the literature, all compounds have been previously identified in *S. officinalis* [17,18,19,20,21].

Compound **3**, with a retention time of 8.84 min and [M-H]^−^ of 1265.5768^2−^, revealed fragment ions at *m*/*z* values of 1103.5236, 1085.5148, 779.2445, 617.1937, 485.3257, and 125.0233. Based on its molecular weight and fragmentation pattern, which were compared with the previously described values of fragment ions of saponarioside C and saponarioside D [21], it was identified as saponarioside C and saponarioside D. Compound **2** (retention time 8.58) was identified as saponarioside E, with a molecular ion [M-H]^−^ having an *m*/*z* value of 1295.5892 and fragment ions at *m*/*z* values of 1133.5363, 1115.5216, 953.5021, 809.2554, and 485.3260, as were previously reported in the literature [21]. Compounds **1**, **4**, **5**, and **6** demonstrated a fragmentation pattern with product ions at *m*/*z* values of 939.4517 and 469.3272, which are characteristic of the gypsogenin (G) backbone, as documented in the literature [17].

Quantification analysis also revealed that the major saponin components of the extract were the G hexasaccharide (compound **6**; *m*/*z* of 1447.6343) and the G octosaccharide (compound **5**; *m*/*z* of 1683.7226) at 0.554% and 0.314%, respectively (Table 2).

### 2.2. Identification and Quantification of Phenolic Compounds in S. officinalis

The total phenolic content (TPC) of methanol, ethanol, and acetone root extracts of *S. officinalis* was detected using the Folin–Ciocalteu method and a standard curve of gallic acid, as previously described [23]. All results were expressed as mg gallic acid equivalents per gram of crude extract (mg GAE/g). According to the data collected, the acetone extract demonstrated the highest TPC result (17.813 mg GAE/g crude extract), which was a yield significantly higher than the methanol and ethanol extracts (*p* < 0.01) (Table 3).

The phenolic compounds identified in the acetone *S. officinalis* root extract, using UHPLC-QTOF-MS/MS, are presented in Table 4, and the total ion chromatogram is documented in the Supplementary Material (Figure S2). The MS/MS fragmentation pattern and chromatograms of all identified compounds are also provided in the Supplementary Material (Figure S3). Six phenolic compounds were identified, including rutin, quercetin galactoside, syringic acid, apigenin, protocatechuic, and vanillic acid. The structural identification of these compounds was based on comparing their MS/MS data with those reported in the literature [24,25].

More specifically, compound **1** (retention time, 4.23 min), which demonstrated an *m*/*z* of 329.0880, was assigned as vanillic acid O-hexoside based on three main fragment ions at *m*/*z* values of 209.0451, 167.0352, and 123.0451 [24]. Compound **2** generated an [M-H]^−^ ion at an *m*/*z* value of 359.0949 (C_15_H_20_O_10_), and in the secondary mass spectrum, it produced fragment ions at *m*/*z* values of 290.0747, 197.0455, 153.0558, and 95.0128. According to data in the literature, this compound has been identified as syringic acid O-hexoside [8]. Compound **3**, with a generated formula (C_13_H_16_O_9_) and an [M-H]^−^ ion at an *m*/*z* of 315.0724, produced two fragment ions at *m*/*z* values of 225.0408 and 152.0114 and was identified as protocatechuic acid based on previously reported data [25]. Compound **4** (retention time of 6.39 min and *m*/*z* of 563.1402) gave three fragmentation product ions at *m*/*z* values of 413.0869, 293.0449, and 89.0240. By comparing these results to previously reported data [24], this compound was identified as apigenin. Compound **5**, with a generated formula C_27_H_30_O_16_, retention time of 6.64 min, and *m*/*z* of 609.1462, gave characteristic product ions, as documented in Table 4 and Figure S3. A comparison of these data to the literature [24] suggests that this compound was rutin. Finally, compound **6** (retention time of 6.93 min), which generated an ion [M-H]^−^ of 463.0882 and produced fragment ions at *m*/*z* values of 300.0278 and 178.9992 (Table 4), was identified as Quercetin 3-O-galactoside [24].

Quantification analysis revealed that among the phenolic compounds identified, protocatechuic acid (compound **3**) was the major constituent detected at 0.217% (Table 4).

### 2.3. Antioxidant Activity of S. officinalis Root Extracts

Further to their phenolic compound content, *S. officinalis* extracts were examined for their antioxidant activity and compared to the known antioxidant standard Trolox. The results are expressed as the half-maximal inhibitory concentration (IC_50_), which is defined as the concentration of each sample (mg/mL) required to scavenge the 2,2-diphenyl-1-picrylhydrazyl (DPPH) radical by 50%. The Trolox equivalent antioxidant capacity (TEAC) was also calculated to determine the antioxidant capacity, as previously described [8]. According to the results, *S. officinalis* ethanol and acetone extracts demonstrated similar TEAC values (2.047% and 2.743%, respectively), whereas the methanol extract was far lower (0.113%, *p* < 0.05) (Table 5).

**Figure 1:**
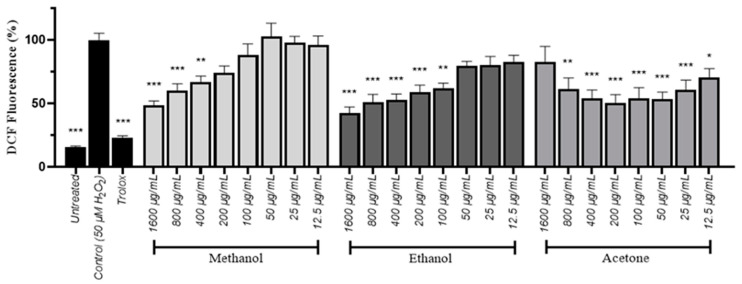
The antioxidant potential of *S. officinalis* root extracts in SH-SY5Y cells in response to H_2_O_2_-induced oxidative stress. The standard error of the mean for five independent DCFDA assays is depicted by error bars; *, **, and *** designate statistical importance at *p* < 0.05, *p* < 0.01, and *p* < 0.001, correspondingly, against control cells that were incubated with 50 μM H_2_O_2_.

For further investigation of the antioxidant activity of *S. officinalis* root extracts, a 2’-7’-Dichlorodihydrofluorescein diacetate (DCFDA) assay was performed in human neuroblastoma (SH-SY5Y) cells to assess the ability of the extracts to reduce free radicals’ levels in cells treated with hydrogen peroxide. The methanol and ethanol extracts were most efficient at higher concentrations, while their antioxidant ability faded in lower concentrations. Conversely, the acetone extract demonstrated its maximum antioxidant capacity within the 50–400 μg/mL range, thus diminishing at lower and higher doses (Figure 1). The results were comparable to the DPPH assay, as shown by the IC_50_ values. The IC_50_ in DCFDA corresponds to the concentration of extract required to decrease by 50% the presence of radical oxygen species (ROS) in cells. The acetone extract was the most prominent, since it presented the lowest IC_50_ value, followed by the ethanol extract, while the methanol one exhibited a weak antioxidant capacity (Table 5).

### 2.4. Antimicrobial Activity of S. officinalis Root Extracts

The minimum inhibitory concentration (MIC) and minimum bactericidal concentration (MBC) of methanol, ethanol, and acetone extracts of *S. officinalis* root extracts were evaluated against Gram-negative bacteria (*Escherichia coli* and *Salmonella enteritidis*) and Gram-positive bacteria (*Staphylococcus aureus* and *Enterococcus faecalis*). As shown in Table 6, all *S. officinalis* extracts (methanol, ethanol, and acetone) demonstrated bacterial inhibition with MIC values ranging from 1.56–3.12 mg/mL for *S. aureus* and *S. enteritidis* and 3.12–6.25 mg/mL for *E. faecalis*. The MIC value for *E.coli* was 3.12 mg/mL, regardless of the solvent used. The antimicrobial efficacy was also studied by determining the MBC, which is defined as the lowest bactericidal extract concentration. Therefore, the lower the MBC value, the less extract is needed to kill the bacteria. *S. officinalis* exhibited MBC values ranging from 3.12–6.25 mg/mL for *S. aureus* and 6.25–12.50 mg/mL for *E. faecalis*. The MBC values against *E. coli* and *S. enteritidis* were the same for *S. officinalis*, regardless of the type of extract solvent used.

### 2.5. Cytotoxicity of S. officinalis Extracts on Cancer Cell Lines

This assay evaluated the cytotoxic effect of *S. officinalis* root extracts—methanol, ethanol, and acetone—on human malignant melanoma (A375) and human cervical epithelioid carcinoma (HeLa) cells. Cancer cells and healthy human keratinocytes (HaCaTs) were treated with increasing concentrations (0–200 μg/mL) of *S. officinalis* root extracts for 72 h. The inhibitory concentrations (IC_50_) for each extract were also calculated and are presented in Table 7.

**Figure 2:**
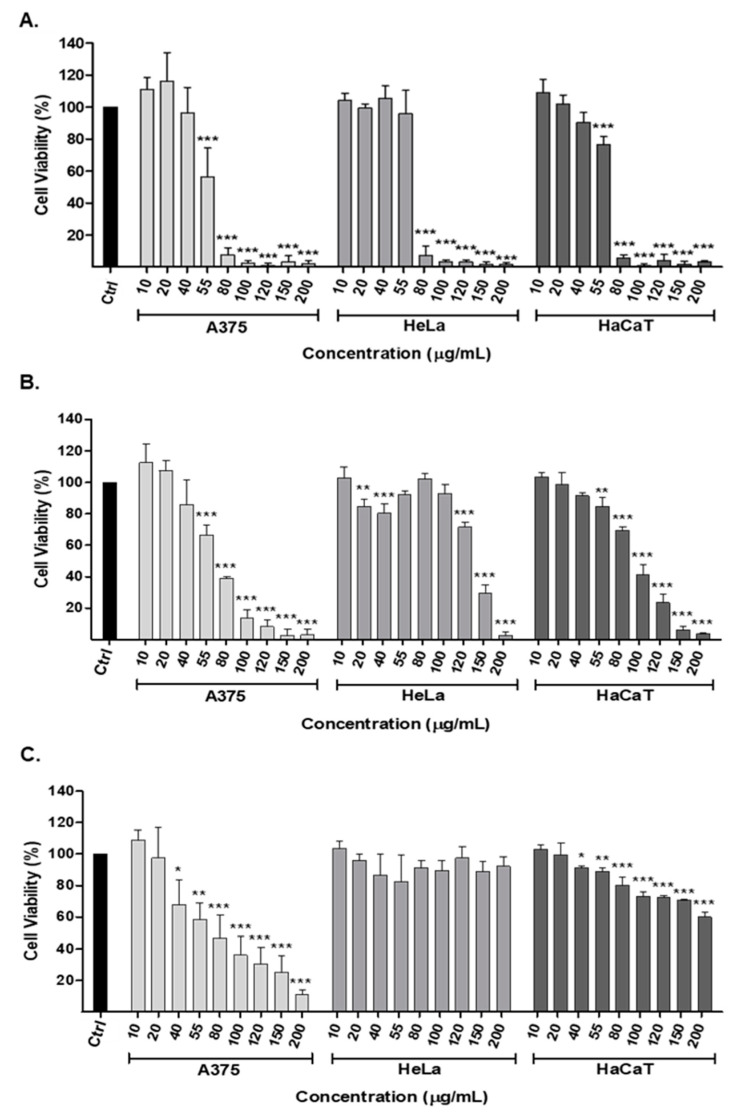
Cytotoxic effects of *S. officinalis* root extracts ((**A**) methanol, (**B**) ethanol, and (**C**) acetone extract) on A375, HeLa, and HaCaT cell lines. A375: human malignant melanoma; HeLa: human cervical epithelioid carcinoma; HaCaT: human keratinocyte. Cells were treated with or without extracts in different concentrations (0–200 μg/mL) for 72 h and evaluated by alamarBlue assay. All data are presented as mean values ± standard deviation and are representative of three independent experiments. *p* values * < 0.05, ** < 0.01, and *** < 0.001 compared to control.

According to our data, the methanol (Figure 2A) and ethanol (Figure 2B) extracts exhibited cytotoxicity against both cancer and healthy cells, with the former demonstrating lower IC_50_ values in all three cell lines tested (Table 7). Specifically, the corresponding methanol versus ethanol IC_50_ values were 55.07 ± 4.42 vs. 62.71 ± 4.88 μg/mL for A375, 67.46 ± 7.32 vs. 137.5 ± 0.88 μg/mL for HeLa, and 61.47 ± 1.62 vs. 92.98 ± 3.49 μg/mL for HaCaT cells. Contrary to the methanol and ethanol extracts, *S. officinalis* acetone displayed cytotoxicity selectively against the A375 cell line (Figure 2C), with a significantly reduced IC_50_ value compared to healthy human keratinocytes (67.01 ± 22.16 vs. 268.93 ± 20.31 μg/mL, *p* < 0.001). The acetone extract had no cytotoxic effect on the HeLa cell line (the IC_50_ was not determined).

### 2.6. Neuroprotective Activity of S. officinalis Extracts

The potential of saponins and phenolics to act as anti-Alzheimer’s compounds has been documented in the literature [26,27]. For this, our extracts were tested as to whether they could ameliorate the toxicity caused by amyloid beta 25–35 (Aβ_25–35_) peptides in SH-SY5Y human neuroblastoma cells. Initially, we investigated the potential cytotoxic effect of the extracts to define their maximum nontoxic concentrations in SH-SY5Y cells. Similarly to the cytotoxicity experiments in A375, HeLa, and HaCaT cell lines, the most cytotoxic extracts were ethanol (≥50 μg/mL) and methanol (≥100 μg/mL). The acetone extract was the least toxic, thus exhibiting cytotoxicity at ≥400 μg/mL (Figure S5).

Then, the possible anti-neurotoxic potential of the *S. officinalis* extracts was evaluated by incubating SH-SY5Y cells with the neurotoxic Aβ_25–35_ peptides, which reduced cell viability by around 45%. Pretreatment of these cells with the three *S. officinalis* extracts, in various non-cytotoxic concentrations just below their maximum nontoxic values, did not significantly reverse the cytotoxic effects of Aβ_25–35_ (Figure S6).

## 3. Discussion

The present study aimed to document the saponin and phenolic chemical profile of *S. officinalis* root extracts. Furthermore, the root extracts’ antioxidant, antibacterial, and anticancer activity were also investigated.

Three different solvents were used to prepare the root extracts (methanol, ethanol, and acetone). The total saponin content extracted with these solvents showed that acetone exhibited the highest yield (17 mg/g crude extracts), and this result agrees with previously reported data [17]. Saponins, as secondary metabolites synthesized by plants, consist of a polar and nonpolar aglycone coupled with saccharide moieties. This combination of polar and nonpolar molecules in their structure may contribute to the higher extraction yield of saponins using an acetone solvent than a methanol and ethanol solvent [3]. The saponin content of *S. officinalis* acetone root extract seems to be lower when compared to the corresponding content previously reported for the *Saponaria cypria* acetone root extract [8]. This may be attributed to the fact that we are dealing with different *Saponaria* species, or it may be due to variations in environmental and growth conditions, as previously reported [28].

The chemical composition and identification of saponins in the root of *S. officinalis* have been previously reported in the literature [17,18,19,20,21,22]. These studies have demonstrated the identification of six major saponins, including gypsogenin and gypsogenic acid derivatives, as well as the saponariosides C, D, and E, which, according to Budan et. al. and Jia et. al., are characteristic of the plants of the *Saponaria* genus [17,21]. Quantitative analysis of the root extract of *S. officinalis* was performed for the first time in this study. According to the results, the gypsogenin octo- and hexasaccharide derivatives are present in higher quantities than the other saponin compounds identified. These results agree with previously published data on the quantification analysis of the root extracts of *S. cypria* [8]. Consequently, it has been confirmed that *Saponaria* species are a good source of saponins, and we demonstrate that gypsogenin derivatives are the most common saponins present in *S. officinalis.* Gypsogenic acid derivatives have been previously reported to possess antimicrobial activity against several bacterial pathogens [29]. Gypsogenin identified in *S. officinalis* roots has also been documented to have cytotoxic properties by several mechanisms, such as inducing apoptosis and activating caspase in cancer cells [20]. Thus, the antibacterial and anticancer activity of *S. officinalis* root extracts demonstrated in this study may be associated with the high concentration of gypsogenin saponin derivative molecules.

The present study has demonstrated the antibacterial activity of *S. officinalis* root extracts against all four strains tested, namely *E. coli*, *S. aureus*, *E. faecalis*, and *S. enteritidis*. The antibacterial inhibition of *S. officinalis* methanol extracts against *S. aureus* and *E. faecalis* has been previously reported in the literature [10,15]. According to the experiments performed by Eren et. al, *S. officinalis* gave higher MIC values, thus demonstrating a lower antibacterial activity compared to the MIC data of this study [16]. However, these differences may be attributed to the different methodologies used. The antimicrobial properties of other *Saponaria* species have also been previously reported. More specifically, methanol, ethanol, and acetone root extracts of *S. cypria* were reported to demonstrate antibacterial activity against *S. aureus* and *E. faecalis* [8]. Similarly to this study, the acetone extract of *S. cypria* demonstrated the highest inhibition against Gram-positive bacteria, with the MIC values being significantly lower than those observed with *S. officinalis* acetone root extracts. Other studies reported the antimicrobial effect of *Quillaia saponaria* saponins on the growth of *E. coli* [30]. Consequently, plants that contain significant levels of saponins seem to strongly impede the growth of several bacterial species.

The presence of polyphenolic compounds in several plants has been linked to the antioxidant and antibacterial activity demonstrated by these plants. Natural antioxidants exhibiting a wide range of biological effects have been demonstrated to protect against oxidative stress and limit the risk of various degenerative diseases [31]. Therefore, this study also aimed to investigate the presence of phenolics in *S. officinalis* root extracts. The results indicate that *S. officinalis* roots are a source of polyphenolics. More specifically, a total of six phenolic compounds were identified. The extracts presented high amounts of protocatechuic acid, a major plant metabolite derived from anthocyanin, which has been found to demonstrate antibacterial activity against *S. aureus* [32] and antioxidant activity using the DPPH and 2,2′-azino-bis(3-ethylbenzothiazoline-6-sulfonic acid (ABTS) methods [33]. Other phenolics were detected at lower concentrations, including quercetin galactoside, vanillic acid derivative, and syringic acid hexoside. Quercetin, a typical representative of flavonols found in several plants, is well known for its antimicrobial activity against Gram-positive and Gram-negative bacteria, as well as for its strong radical scavenging activity [34]. Rutin, another phenolic compound identified in this study, has been reported to have diverse pharmacological activities due to its high antioxidant properties [35], thus protecting from oxidative stress. Apigenin, a naturally occurring flavone identified in this study, has been reported to demonstrate antimicrobial activity against *S. aureus*-resistant bacteria with a MIC concentration similar to that of quercetin [36], as well as other health-related effects such as the prevention of oxidative damage caused by reactive oxygen molecules [37]. Even though *S. officinalis* has been previously reported to contain phenolics, this is the first study that identified the type of flavonoids in root extracts and quantified their concentration. The fact that these extracts are a good source of phenolic compounds may contribute to their important antioxidant and antimicrobial role.

In addition to their recognized antioxidant, antibacterial, and antimicrobial properties, certain chemical compounds identified in *S. officinalis* root extracts have also been previously reported for their potential anticancer activity [38,39,40]. For instance, quercetin is renowned for its bioactive polyphenolic nature, and it exhibits varied biochemical and pharmacological functions resulting from the distinct arrangement of its functional groups. It could be present in two different forms: the free-state or aglycone form [38,41] (e.g., quercetin galactoside, as identified in this study), which are responsible for its antioxidant properties and its ability to inhibit several types of human cancer, such as breast, lung, nasopharyngeal, prostate, ovarian, pancreatic, and leukemia, through diverse mechanisms [40,41,42,43,44,45,46,47,48]. Specifically, the cellular mechanisms quercetin uses to inhibit various cancers include the induction of apoptosis or cell cycle arrest, antioxidant actions, and the downregulation of cancer-related proteins [41]. Vanillic acid, syringic acid, and apigenin, present in *S. officinalis* extracts, exhibit significant anticancer activity. Their anticarcinogenic effects are associated with their capability to inhibit cell proliferation through several molecular pathways, thus inducing apoptosis and cell cycle arrest across various cancer cell types [38,39]. Therefore, it was imperative to investigate the potential anticancer effects of *S. officinalis* extracts on various cancer cell lines.

The findings from this study revealed that, among the three types of extracts tested, methanol and ethanol extracts exhibited the highest cytotoxicity, thus affecting both cancer and healthy cells alike. Conversely, the acetone extract of *S. officinalis* demonstrated selective cytotoxicity against A375 cells, thus inducing a significant reduction in cell viability in a concentration-dependent manner while showing minimal impact on the healthy HaCaT cell line. The specific growth inhibition observed against certain cancer cell lines corresponds to the superior antioxidant activity exhibited by the acetone extract, as evidenced by both the DPPH and DCFDA assays, compared to the methanol and ethanol extracts. Considering the above information for certain phenolic compounds, these results suggest the promising potential of the acetone extract for anticancer activity and anticancer resistance in specific human cancer cell lines.

There is no documented evidence regarding the anti-Alzheimer’s disease potential of *Saponaria* species. The presence of saponins like gypsogenin [49] and phenolics such as protocatechuic acid, apigenin, quercetin, and rutin—molecules known for their neuroprotective properties [27,50,51]—motivated us to evaluate the extracts’ anti-neurotoxic activity against Aβ_25–35_ toxicity. No such significant action was found, which was probably due to antagonistic interactions between the extracts’ compounds that may impair certain biological activities of the individual molecules.

To validate the aforementioned findings, it is essential to conduct supplementary experiments aiming to explore the anticancer potential of *S. officinalis* extracts across a broader spectrum of human cancer types. Additionally, these experiments should uncover the cellular mechanisms underlying the inhibition of cancer proliferation. Further experiments focused on cellular mechanisms, such as assessing cell viability, analyzing the cell cycle, and monitoring the expression of pro-apoptotic molecules and metabolites, are integral steps in further elucidating the anticancer properties of *S. officinalis* extracts.

## 4. Materials and Methods

### 4.1. Preparation of the Extracts

Plant roots were collected from 15 randomly selected mature *S. officinalis* plants (total dry root mass = 500 g), cultivated at the nurseries of the Department of Forests in Cyprus. Cultivated plants came from seeds germinated at the Nature Conservation Unit at Frederick University. Seeds were bought from Jelitto Staudensamen GmbH in 2019 (Code No. SG144). *S. officinalis* plants were identified and distinguished from other *Saponaria* species occurring in Cyprus based on morphological characteristics [52]. *S. officinalis* roots were washed, air-dried and crushed into fine powder. Ten gr of root powder was extracted each time with 150 mL of a different solvent (methanol, ethanol, or acetone) and macerated for 24 h. Afterwards, the extracts were centrifuged at 4 °C, 4000 rpm for 10 min and filtered. The solvent in each extract was fully evaporated using a rotary evaporator (Stuart RE300, Keison, Chelmsford, UK) at 40 °C under vacuum of <1 mmHg according to the protocol previously described [8]. The root crude extracts were stored at 4 °C until further analysis.

### 4.2. UHPLC-QTOF-MS Analysis

The identification of the saponin components and the phenolic compounds was performed using UHPLC-QTOF-MS (Agilent Technologies, Santa Clara, CA, USA) analysis with a method as previously described in Charalambous et al. [8]. The molecular formula assignment was carried out for each identified compound by comparing the experimental to theoretical *m*/*z* values to have a mass deviation below 5 ppm. The molecular weight values and the fragmentation pattern of the compounds were compared to previously reported values of signature ion fragments of known saponins [10,26] and phenolics [28,29]. Relative quantification was based on calculated peak areas of the six saponins using the linear regression response curve of reference for quillaic acid (Sigma Aldrich, Darmstadt, Germany). Similarly, the linear regression response curve of reference for quercetin (Sigma Aldrich, Germany) was used for the quantification of the six phenolic compounds. The standard concentration range used for quantification was 5, 10, 50, 100, 200, 400, 600, and 800 ng/injection for quillaic acid and 5, 10, 50, 100, 200, and 400 ng/injection for quercetin. The data are presented as the mean % (g of compound per 100 g of crude extract) ± the estimated standard deviation (SD) of three independent experiments.

### 4.3. Total Saponin Content

The total saponin content of *S. officinalis* root extracts was measured as previously described [8]. All results are expressed as mg of oleanolic acid equivalents per gram of crude extract (mg OAE/g crude extract) based on oleanolic acid calibration curve (linear regression: 0.0025–0.25 mg/mL; R^2^ > 0.9946).

### 4.4. Total Phenolic Content

The total phenolic content of *S. officinalis* root extracts was determined using the Folin–Ciocalteu method and a standard gallic acid curve (linear regression: 0.10–0.4 mg/mL; R^2^ > 0.9950) as previously described [8]. All results were expressed as mg of gallic acid equivalents per gram of crude extract (mg GAE/g crude extract).

### 4.5. 2,2-diphenyl-1-picrylhydrazyl (DPPH) Assay

The antioxidant activity of *S. officinalis* root extracts was determined using the DPPH free radical scavenging assay as previously described [8]. DPPH solution (0.5 mM in 100% methanol) was prepared, and 100 μL of this solution was added to different concentrations of the extracts (0.097–50 mg/mL). The mixture was allowed to stand for a 30 min incubation period at room temperature in the dark. Finally, the absorbance at 515 nm was recorded using a microplate reader (Sunrise, Tecan Trading Ltd., Mannedorf, Switzerland). A total of 1 mM Trolox was used as a reference sample. The antioxidant activity of the extracts was calculated as a percentage of the scavenging activity of DPPH solution using the following equation:

DPPH Scavenged (%) = ((AB–AA)/AB) × 100

(AB is the absorbance of control sample; AA is the absorbance of the sample at 30 min).

The half-maximal inhibitory concentration (IC_50_) was defined as the concentration of the extracts (mg/mL) required to scavenge the DPPH radical by 50%. The Trolox equivalent antioxidant capacity (TEAC) of the extracts was calculated as follows:

TEAC = IC_50_ of Trolox (mg/mL)/IC_50_ of sample (mg/mL)

### 4.6. Dichlorofuoresence Diacetate (DCFDA) Assay

To assess the antioxidant potential of the *S. officinalis* extracts, the presence of reactive oxygen species was evaluated using the 2′,7′–dichlorofluorescin diacetate (DCFDA) assay as previously documented by Ververis et al. [53]. SH-SY5Y cells were cultured for 24 h at a density of 2.5 × 10^5^ cells/well in a black 96-well plate. Following 45 min of treatment with 20 μM DCFDA solution in a humidified incubator at 37 °C, cells were treated with various concentrations of extract and 50 μM hydrogen peroxide. Fluorescence at Ex/Em = 485/535 nm was recorded in a Synergy H1 microplate reader (BioTek^®^ Instruments, Inc., Winooski, VT, USA). Before calculating the percentage of fluorescence intensity relative to cells treated with hydrogen peroxide only, background fluorescence was subtracted from each value. Trolox (500 μΜ) was employed as a standard antioxidant. Four independent experiments were performed.

### 4.7. Antimicrobial Activity

Broth microdilution method was used for the determination of MIC and MBC of the *S. officinalis* root extracts with a method previously described [8]. The MIC of each extract was defined as the minimum sample concentration that prevented the color change of the medium, thus exhibiting complete inhibition of bacterial growth as compared to the control. The MBC of each extract was defined as the lowest concentration of each sample that did not exhibit a color change after the addition of INT, as described in the literature [23].

### 4.8. Cell Culture

The DCFDA assay was carried out using human neuroblastoma (SH-SY5Y) cells (Leibniz Institute DSMZ, Braunschweig, Germany) cultivated in Dulbecco’s modified Eagle’s medium (DMEM) supplemented with 10% fetal bovine serum, 50 U/mL penicillin, and 50 mg/mL streptomycin (Biosera, Nuaille, France). The cytotoxicity assay was carried out on immortalized human cervical epithelioid carcinoma (HeLa) cells, human malignant melanoma (A375, provided by Prof. Michail Panagiotidis) cells, and healthy human keratinocytes (HaCaTs, provided by Prof. Michail Panagiotidis). HeLa, A375, and HaCaT cells were maintained in DMEM medium (Gibco, Thermo Fisher Scientific, Waltham, MA, USA) supplemented with 10% fetal bovine serum (FBS) (Gibco, Thermo Fisher Scientific, Waltham, MA, USA), 1% penicillin/streptomycin solution (Gibco, Thermo Fisher Scientific, Waltham, MA, USA) and 4 mM L-glutamine (200 nM) (Gibco, Thermo Fisher Scientific, Waltham, MA, USA). All the cells were cultured in a humidified atmosphere in a 5% CO_2_ incubator at 37 °C.

### 4.9. Preparation of S. officinalis Extracts for CytotoxicityAssay (alamarBlue Assay)

The three *S. officinalis* root extracts [methanol (MEOH), ethanol (ETOH), and acetone (ACE)] were dissolved in dimethyl sulfoxide (DMSO) and were diluted in a fresh complete medium before each experiment. The final concentration of DMSO for cell treatment was 0.4%. The solution of each extract was stored at 4 °C until required.

### 4.10. Cytotoxicity Assay (alamarBlue Assay)

The antiproliferative and cytotoxic activity of prepared *S. officinalis* root extracts were tested against human malignant melanoma (A375), human cervical epithelioid carcinoma (HeLa), and human keratinocyte (HaCaT) cell lines using alamarBlue assay as described by Kyriakou et al. [54]. The cells (A375; 1.5 × 10^3^, HeLa; 8 × 10^3^, and HaCaT; 5 × 10^3^ cells/100 μL) were seeded in 96-well plates and incubated for 24 h. After that, a fresh medium containing different concentrations of the tested extracts of *S. officinalis* (0–200 μg/mL) was added. The incubation lasted 72 h. Control cells were incubated with either complete medium only and/or DMSO (0.4%). Each sample was running out in triplicate in a final volume of 200 μL. At the indicated time point, resazurin dissolved in PBS was added in an amount equal to 1/10 (0.05 mg/mL final concentration) of the final volume in each well and incubated for 4 h at 37 °C. The absorbance at 570 nm and 600 nm (as a reference wavelength) wase measured using Synergy H1 microplate reader (BioTek^®^ Instruments, Inc., Winooski, VT, USA). Cytotoxic activity was assessed based on cell viability expressed as a percentage of control cells (0 μg/mL). Results were means of three independent measurements (±standard deviation: SD). The IC_50_ values were determined by plotting the percentage viability of the cells versus concentration, and the adequate calculation was made using Excel and GraphPad Prism (version 5.0, GraphPad Software).

### 4.11. Peptides Preparation

Aβ_25–35_ peptides (Genscript, Piscataway, NJ, USA) were dissolved in sterile distilled water at a concentration of 1 mM and incubated for one week at 37 °C. Then, the peptides were aliquoted and stored at −20 °C.

### 4.12. Assessment of Neuroprotective Activity and MTT Assay

The MTT assay evaluated the SH-SY5Y cell viability after treatment with *S. officinalis* extracts, Aβ_25–35_ peptides, or the combination of both extracts and peptides, as previously described [50]. The SH-SY5Y cells were cultured for 24 h at a density of 2 × 10^5^ cells/well in a 96-well plate. The next day, the cells were treated with different concentrations of the *S. officinalis* extracts, or 25 μM of Aβ_25–35_ peptides, or a combination of both for 48 h. Then, the cells were incubated in DMEM without phenol red that contained 45 μg/mL MTT for 4 h at 37 °C. Following aspiration of the medium, 150 μL of DMSO was added to each well. After covering the plate with foil and shaking it for 15 min, the absorbance was measured at 590 nm using a Synergy H1 microplate reader (BioTek^®^ Instruments, Inc., Winooski, VT, USA). Cell viability was calculated using the following equation:

Cell viability (%) = [(OD treated cells − OD blank)/(OD control − OD blank)] × 100.

(OD is the absorbance)

At least four independent experiments were performed.

### 4.13. Statistical Analysis

The experiments for antioxidant activity were performed in triplicates, and the results are expressed as the mean value ± estimated standard deviation (SD). Data for the DCFDA assay are shown as mean values ± standard error (SEM) of the mean, and statistical analyses were performed using one-way ANOVA with Dunnett’s test for multiple comparisons. Data for the cytotoxic assay are expressed as mean values ± standard deviation (SD), and statistical analyses were performed using one-way ANOVA with Tukey’s test for multiple comparisons. The statistical software GraphPad Prism version 5.0 was used for the statistical analysis, and statistical significance was concluded with *p* > 0.05.

## 5. Conclusions

This study aimed to document the chemical profile and evaluate the biological activities of *S. officinalis* root extracts. Specifically, it focused on identifying the saponin and phenolic content, as well as assessing the antioxidant, antibacterial, and anticancer activities of the extracts prepared using methanol, ethanol, and acetone solvents. The extraction process revealed that acetone was the most effective solvent for saponin extraction. Quantitative analysis identified six major saponins, including gypsogenin and its derivatives. Gypsogenin octo- and hexasaccharide derivatives were found in higher quantities than other saponins. This confirms that *Saponaria* species are a rich source of saponins, with gypsogenin derivatives, which are the most prevalent in *S. officinalis*. The study demonstrated the antibacterial activity of *S. officinalis* root extracts against *E. coli*, *S. aureus*, *E. faecalis*, and *S. enteritidis*. Acetone extracts showed the highest inhibition, particularly against Gram-positive bacteria. *S. officinalis* root extracts were also found to be a good source of polyphenolics, with six phenolic compounds identified. Protocatechuic acid was present in high amounts, which is known for its antioxidant and antibacterial activities. Other phenolics, such as quercetin, rutin, and apigenin, also contributed to the extracts’ antioxidant properties. These compounds are well documented for their ability to neutralize free radicals and protect against oxidative stress, which is linked to various degenerative diseases. Finally, the anticancer potential of *S. officinalis* extracts was also explored. Among the solvents used, methanol and ethanol extracts showed high cytotoxicity against both cancerous and healthy cells. In contrast, acetone extracts exhibited selective cytotoxicity against A375 cancer cells while having minimal impact on healthy HaCaT cells. This selective activity suggests that the acetone extract contains compounds with specific anticancer properties, likely linked to its high antioxidant activity. The anti-neurotoxic activity of the extracts was also evaluated. No significant neuroprotective action was observed against Aβ_25–35_ toxicity, possibly due to antagonistic interactions between the compounds.

In summary, this study provides valuable data on the phytochemical composition and biological activities of *S. officinalis* root extracts, thus paving the way for future research and potential therapeutic applications. Further exploration and validation of these findings could contribute to the development of novel treatments for bacterial infections, oxidative stress-related conditions, and certain types of cancer.

## Figures and Tables

**Table 1:** Total saponin content (TSC) of methanol, ethanol, and acetone root extracts of *S. officinalis*.

Solvent Type	TSC (mg OAE ^1^/g Crude Extract) ± SD
Methanol	9.535 ^a^ ± 0.039
Ethanol	103.117 ^b^ ± 0.466
Acetone	124.635 ^c^ ± 6.277

^1^: mg OAE/g crude extract—mg oleanolic acid equivalents per gram of dry crude extract; SD: standard deviation. ^a–c^: values having different letters differ significantly (*p* < 0.01).

**Table 2:** UHPLC-QTOF-MS mass spectra data in negative ion mode of the major saponin compounds identified in *S. officinalis* root extracts.

Compound Number	RT	Molecular Formula	The *m*/*z* [M-H]^−^	Exp. *m*/*z* [M-H]^−^	Error ppm	MS/MS Product Ions	Compound Name	Saponin Content WT % ± SD	References
**1**	8.47	C_72_H_112_O_37_	1567.6810^2−^	1567.6755^2−^	3.5	1435.6027, 939.4525, 469.3287	G heptasaccharide	0.053 ± 0.011	[17]
**2**	8.58	C_60_H_96_O_30_	1295.5914^2−^	1295.5892^2−^	1.4	1133.5363, 1115.5216, 939.5021, 809.2554, 485.3260	Saponarioside E	0.234 ± 0.027	[21]
**3**	8.84	C_59_H_94_O_29_	1265.5808^2−^	1265.5768^2−^	3.2	1103.5236, 1085.5148, 779.2445, 485.3257, 617.1937, 125.0233	Saponarioside C Saponarioside D	0.040 ± 0.013	[21]
**4**	10.45	C_75_H_118_O_39_	1641.7177^2−^	1641.7145^2−^	2.0	1509.6730, 939.4612, 469.3284	G or GA saccharide	0.207 ± 0.018	[17]
**5**	11.35	C_77_H_120_O_40_	1683.7283	1683.7226	3.4	1551.6807, 939.4567, 469.3284, 113.0238	G octosaccharide	0.314 ± 0.028	[17,22]
**6**	12.44	C_68_H_104_O_33_	1447.6387	1447.6343	3.0	1315.5899, 939.4535, 469.3305, 113.0247, 101.0241	G hexasaccharide	0.554 ± 0.052	[17]

RT: retention time; *m*/*z* [M-H]^−^: value of deprotonated molecule; ^2−^: *m*/2*z* ion detected; ppm: parts per million; WT %: weight percentage (g component per 100 g dry root); SD: standard deviation; G: gypsogenin; GA: gypsogenic acid.

**Table 3:** Total phenolic content (TPC) of methanol, ethanol, and acetone root extracts of *S. officinalis*.

Solvent Type	TPC (mg GAE ^1^/g Crude Extract) ± SD
Methanol	0.159 ^a^ ± 0.077
Ethanol	0.973 ^a^ ± 0.468
Acetone	17.813 ^b^ ± 0.512

^1^: mg GAE/g crude extract—mg gallic acid equivalents per gram of dry crude extract; SD: standard deviation. ^a,b^: values having different letters differ significantly (*p* < 0.01).

**Table 4:** UHPLC-QTOF-MS mass spectra data in negative ion mode of the phenolic compounds identified in *S. officinalis* root extracts.

Compound Number	RT	Molecular Formula	The *m*/*z* [M-H]^−^	Exp. *m*/*z* [M-H]^−^	Error ppm	MS/MS Productions	Compound Name	Phenolic Compounds Content WT % ± SD	Reference
**1**	4.23	C_1_4__H_18_O_9_	329.0878	329.0880	0.6	209.0451, 167.0352, 123.0451	Vanillic acid O-hexoside	0.069 ± 0.016	[24]
**2**	4.54	C_15_H_20_O_10_	359.0984	359.0949	1.3	290.0747, 197.0455, 153.0558, 95.0128	Syringic acid O-hexoside	0.018 ± 0.005	[8]
**3**	5.07	C_13_H_16_O_9_	315.0722	315.0724	0.6	225.0408, 152.0114	Protocatechuic acid	0.217 ± 0.053	[24]
**4**	6.39	C_26_H_28_O_14_	563.1406	563.1402	1.3	413.0869, 293.0449, 89.0240	Apigenin	0.010 ± 0.002	[24]
**5**	6.64	C_27_H_30_O_16_	609.1461	609.1462	0.3	463.0843, 300.0276, 151.0033	Rutin	0.047 ± 0.010	[8]
**6**	6.93	C_21_H_20_O_12_	463.0882	463.0882	0	300.0278, 178.9992	Quercetin 3-*O*-galactoside	0.025 ± 0.007	[8]

RT: retention time; *m*/*z* [M-H]^−^: value of deprotonated molecule; ppm: parts per million; WT %: weight percentage (g component per 100 g dry root); SD: standard deviation.

**Table 5:** Antioxidant activity (IC_50_ and TEAC) of *S. officinalis* root with DPPH and DCFDA assay.

	DPPH Assay		DCFDA Assay
Solvent Type	IC_50_ (mg/mL) ± SD	TEAC (%) ± SD	IC_50_ (mg/mL) ± SD
Methanol	7.517 ^b^ ± 0.222	0.113 ^a^ ± 0.006	0.634 ^c^ ± 0.060
Ethanol	0.423 ^a^ ± 0.025	2.047 ^b^ ± 0.081	0.228 ^b^ ± 0.025
Acetone	0.323 ^a^ ± 0.076	2.743 ^b^ ± 0.509	0.072 ^a^ ± 0.073

IC_50_: half-maximal inhibitory concentration; TEAC: Trolox equivalent antioxidant capacity; DCFDA: 2′,7′–dichlorofluorescin diacetate; DDPH: 2,2-diphenyl-1-picrylhydrazyl; SD: standard deviation, ^a–c^: values with the same letter are nonsignificantly different (*p* < 0.05).

**Table 6:** Minimum inhibitory concentration (MIC) and minimum bactericidal concentration (MBC) for *S. officinalis* root extracts against *E. coli*, *S. aureus*, *E. faecalis*, and *S. enteritidis*.

	*E. coli*			*S. aureus*			*E. faecalis*			*S. enteritidis*			Amp ^1^ (Control)	Gen ^1^ (Control)
	MEOH	ETOH	ACE	MEOH	ETOH	ACE	MEOH	ETOH	ACE	MEOH	ETOH	ACE	-	-
MIC (mg/mL) ± SD	3.12 ± 0.03	3.12 ± 0.03	3.12 ± 0.03	3.12 ± 0.01	1.56 ± 0.04	1.56 ± 0.06	6.25 ± 0.05	3.12 ± 0.03	3.12 ± 0.02	3.12 ± 0.06	3.12 ± 0.10	1.56 ± 0.13	0.004 ± 0.001	0.004 ± 0.002
MBC (mg/mL) ± SD	6.25 ± 0.02	6.25 ± 0.02	6.25 ± 0.02	6.25 ± 0.04	3.12 ± 0.04	3.12 ± 0.03	12.50 ± 0.05	12.50 ± 0.03	6.25 ± 0.02	6.25 ± 0.06	6.25 ± 0.10	6.25 ± 0.13	0.004 ± 0.001	0.008 ± 0.002

^1^: ampicillin and gentamycin were used as control antimicrobial agents against *E. coli*/*S. enteritidis* and *S. aureus*/*E. faecalis*, respectively. Amp: ampicillin; Gen: gentamycin; MEOH: methanol solvent; ETOH: ethanol solvent; ACE: acetone solvent; SD: standard deviation.

**Table 7:** Half-maximal inhibitory concentration (IC_50_) of *S. officinalis* methanol, ethanol, and acetone root extracts against three different cell lines after 72 h of exposure.

	alamarBlue Assay	
Solvent Type	Cell Line	IC_50_ (μg/mL) ± SD
Methanol	A375	55.07 ^a^ ± 4.42
	HeLa	67.46 ^a^ ± 7.32
	HaCaT	61.47 ^a^ ± 1.62
Ethanol	A375	62.71 ^a^ ± 4.88
	HeLa	137.5 ^b^ ± 0.88
	HaCaT	92.98 ^a^ ± 3.49
Acetone	A375	67.01 ^a^ ± 22.16
	HeLa	N/D
	HaCaT	268.93 ^b^ ± 20.31

N/D: not determined; IC_50_: half-maximal inhibitory concentration; SD: standard deviation; A375: human malignant melanoma; HeLa: human cervical epithelioid carcinoma; HaCaT: human keratinocyte, ^a,b^: values with the same letter are nonsignificantly different (*p* < 0.01).

## Data Availability

All the data obtained and materials analyzed in this research are available from the corresponding author upon request.

## Supplementary Materials

The following supporting information can be downloaded at https://www.mdpi.com/article/10.3390/plants13141982/s1. Figure S1: UHPLC-QTOF-MS extracted ion chromatogram of saponins of *S. officinalis* root extract. Only peaks that represent saponins are indicated with numbers 1–6. Other peaks did not provide any evidence that they are saponins; Figure S2: MS/MS spectra data of saponins with precursor and product ions in negative mode; Figure S3: UHPLC-QTOF-MS extracted ion chromatogram of phenolic compounds of *S. officinalis* root extract. Only peaks that represent phenolic compounds are indicated with numbers 1–6. Other peaks did not provide any evidence that they are phenolic compounds; Figure S4: MS/MS spectra data of phenolic compounds with precursor and product ions in negative mode. Figure S5: Neuroprotective activity in SH-SY5Y cells treated with Aβ_25–35_ neurotoxic peptides. Figure S6: Neuroprotective activity of plant extracts in SH-SY5Y cells treated with Aβ_25–35_ neurotoxic peptides.
