# Supplementary figures and images for: Saponin and Phenolic Composition and Assessment of Biological Activities of Saponaria officinalis L. Root Extracts
Source: Plants (Basel). 2024 Jul 19;13(14):1982. doi: 10.3390/plants13141982 (PMC11281274; doi:10.3390/plants13141982)

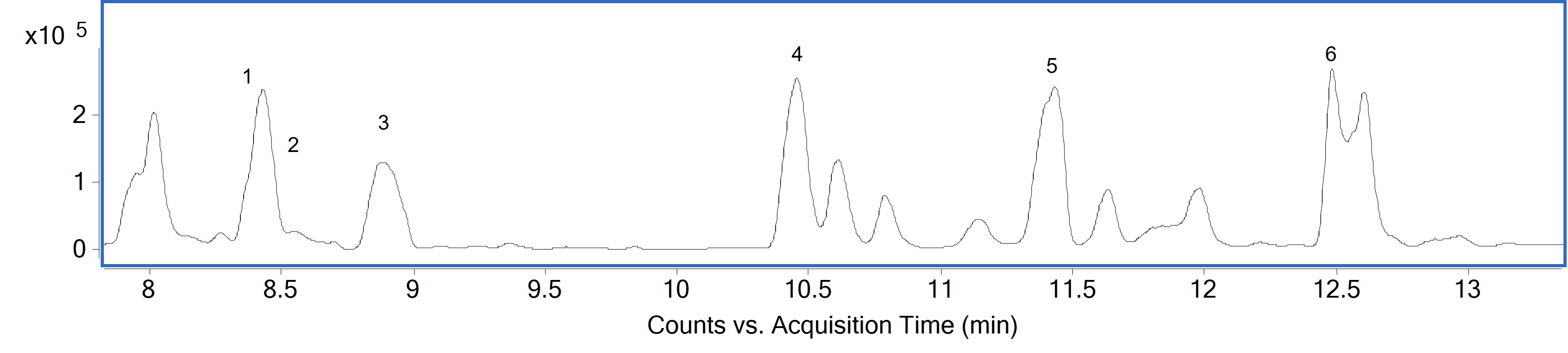

Supplement: Supplementary file 1 [file plants-13-01982-s001.zip › Figure S1 total saponins.jpg]

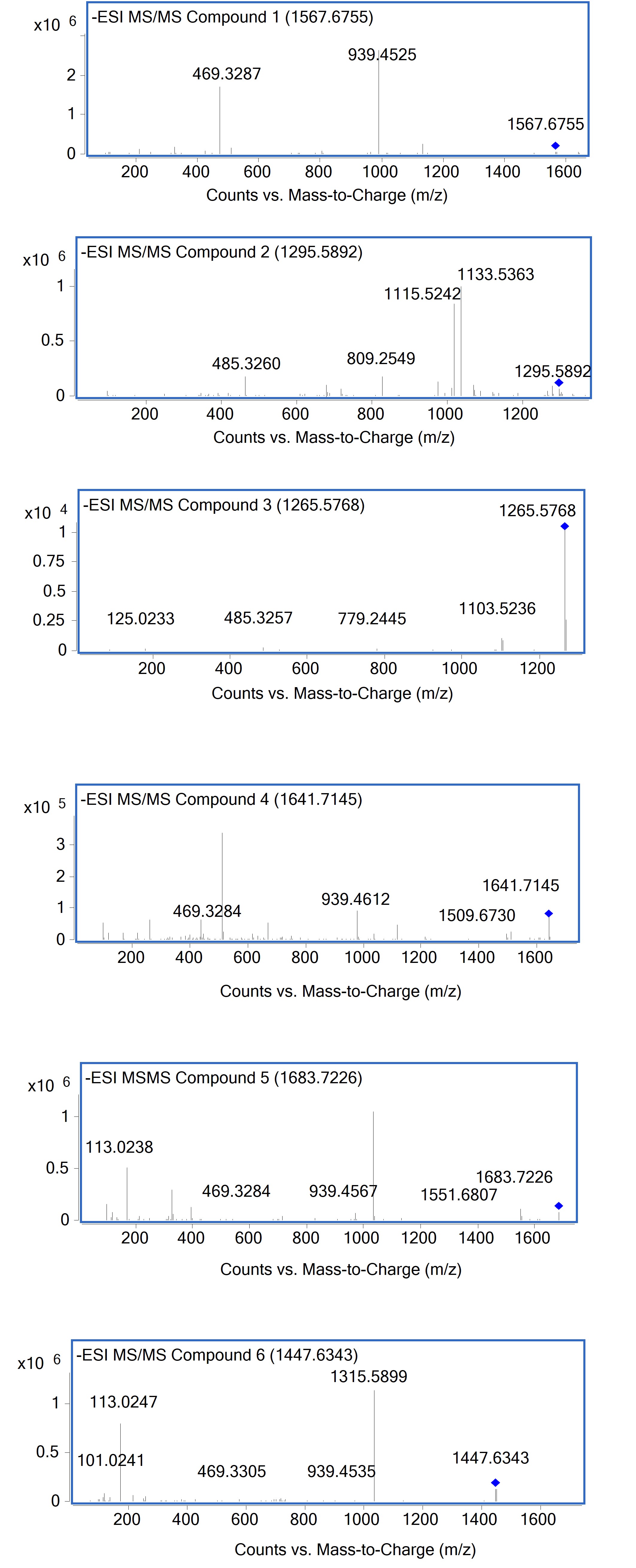

Supplement: Supplementary file 1 [file plants-13-01982-s001.zip › Figure S2 MSMS saponins.jpg]

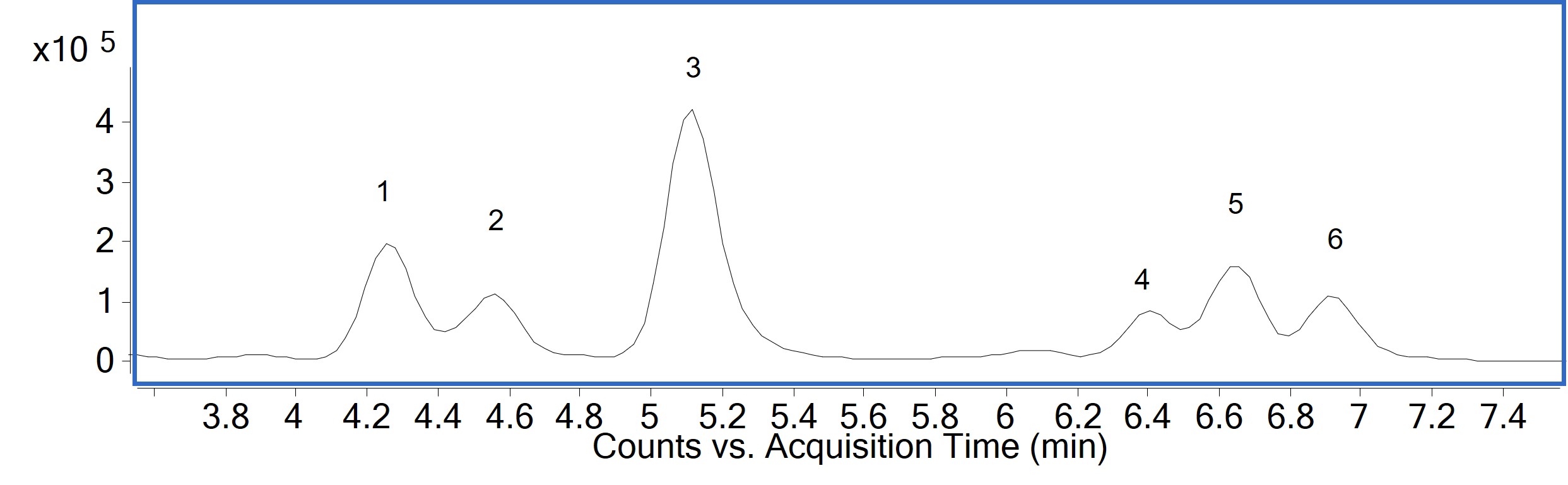

Supplement: Supplementary file 1 [file plants-13-01982-s001.zip › Figure S3 total phenolics.jpg]

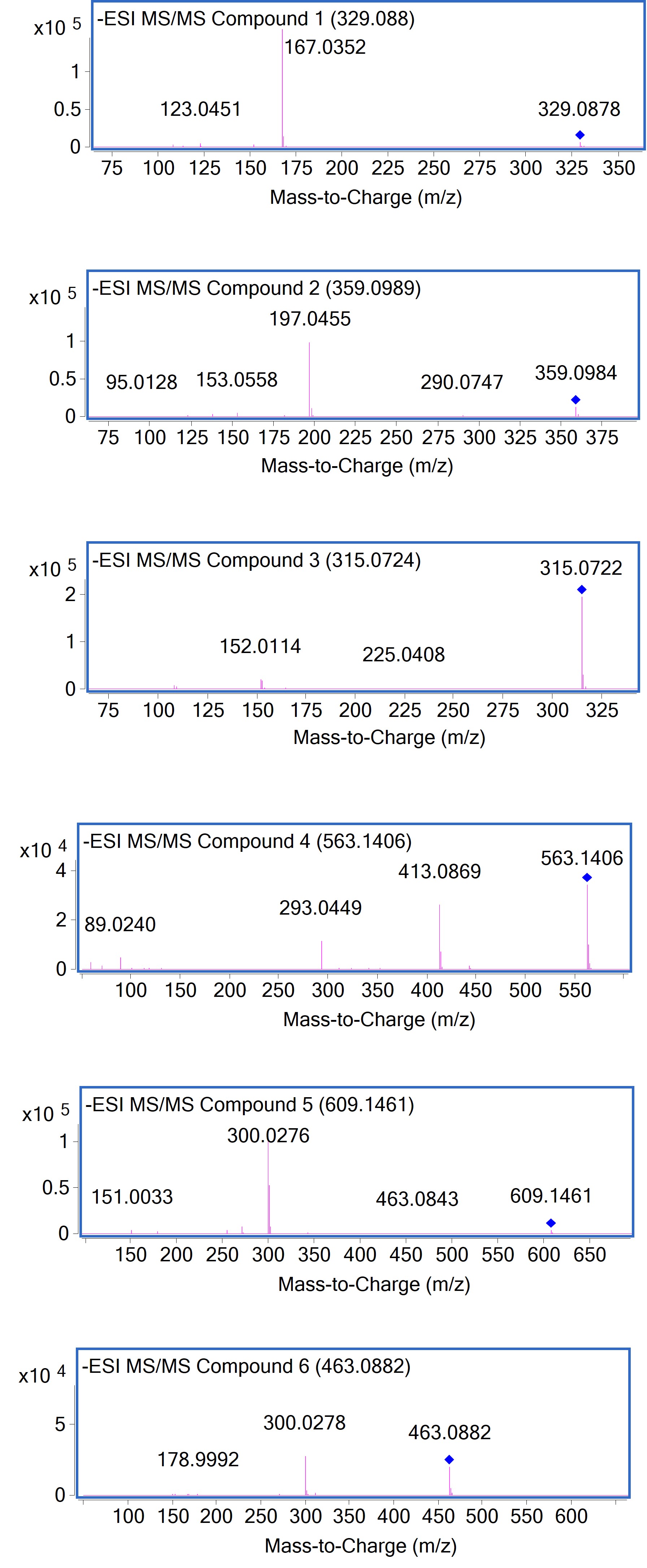

Supplement: Supplementary file 1 [file plants-13-01982-s001.zip › Figure S4 MSMS phenolics.jpg]

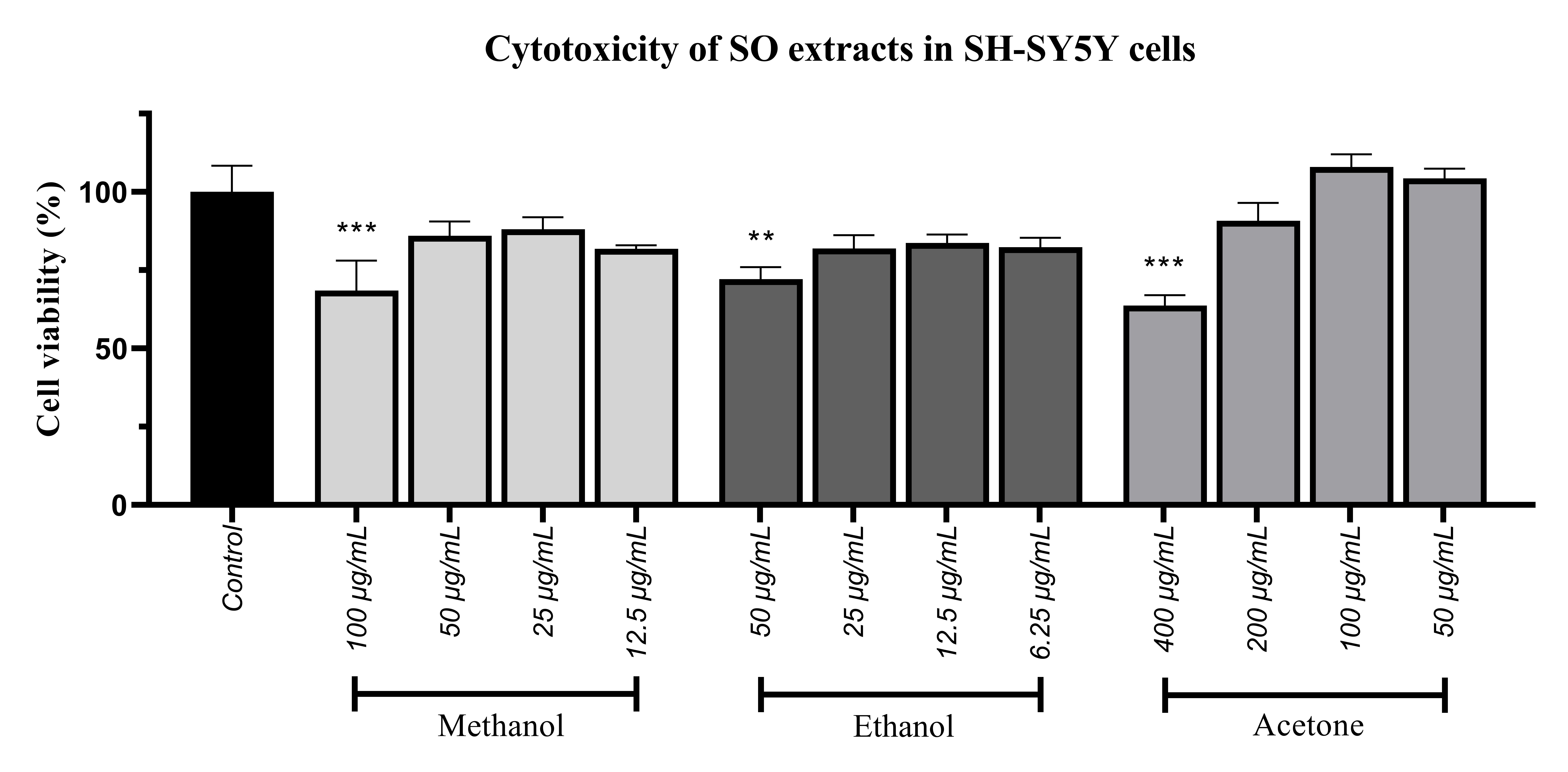

Supplement: Supplementary file 1 [file plants-13-01982-s001.zip › Figure S5 neuroprotective.jpg]

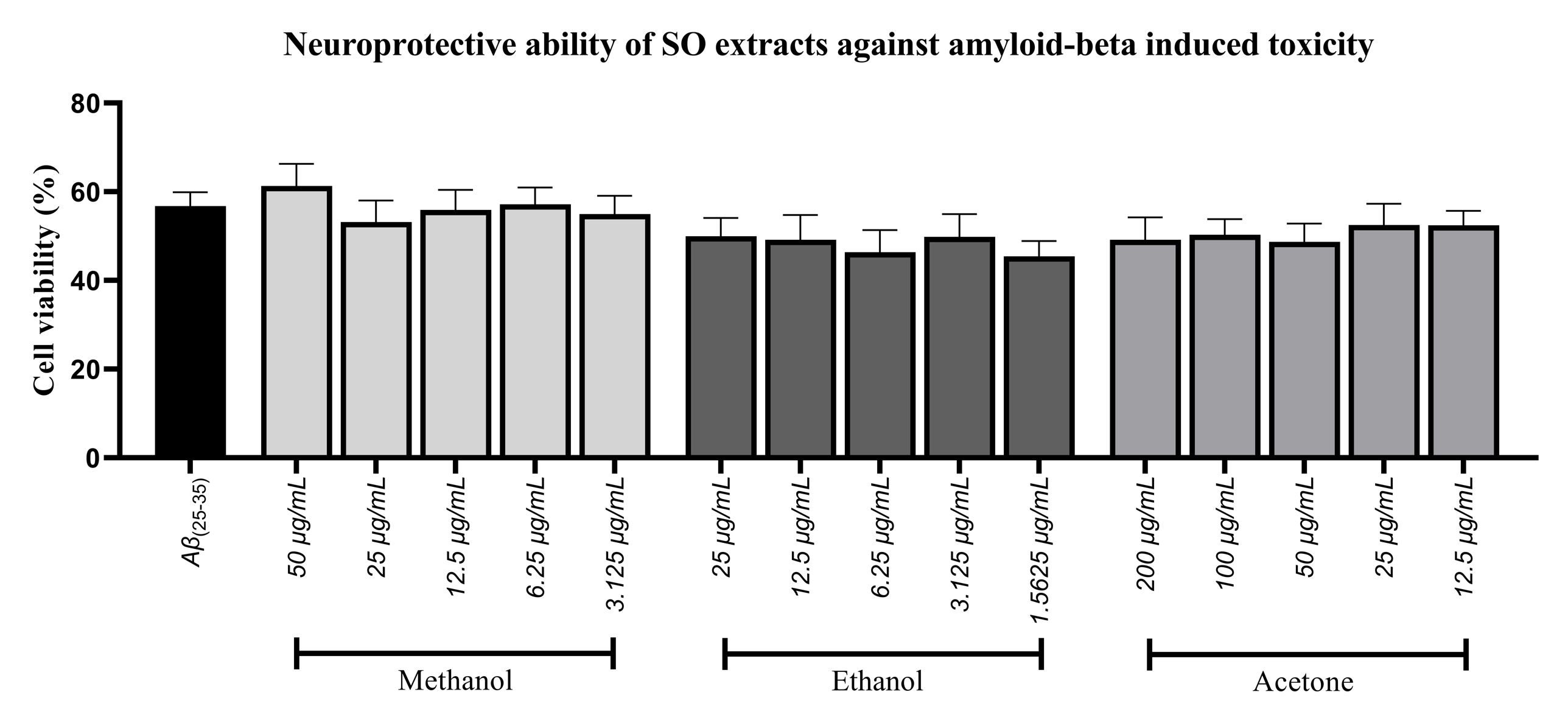

Supplement: Supplementary file 1 [file plants-13-01982-s001.zip › Figure S6 neuroprotective.jpg]
